# Quantification of expected information gain in visual acuity and contrast sensitivity tests

**DOI:** 10.1038/s41598-023-43913-1

**Published:** 2023-10-05

**Authors:** Zhong-Lin Lu, Yukai Zhao, Luis Andres Lesmes, Michael Dorr

**Affiliations:** 1 Division of Arts and Sciences, NYU Shanghai, Shanghai, China; 2 Center for Neural Science and Department of Psychology, New York University, 4 Washington Place, New York, NY 10003 USA; 3 NYU-ECNU Institute of Brain and Cognitive Neuroscience at NYU Shanghai, Shanghai, China; 4 Center for Neural Science, New York University, New York, USA; 5 Adaptive Sensory Technology Inc., San Diego, CA USA

**Keywords:** Psychology, Human behaviour

## Abstract

We make use of expected information gain to quantify the amount of knowledge obtained from measurements in a population. In the first application, we compared the expected information gain in the Snellen, ETDRS, and qVA visual acuity (VA) tests, as well as in the Pelli–Robson, CSV-1000, and qCSF contrast sensitivity (CS) tests. For the VA tests, ETDRS generated more expected information gain than Snellen. Additionally, the qVA test with 15 rows (or 45 optotypes) generated more expected information gain than ETDRS, whether scored with VA threshold alone or with both VA threshold and VA range. Regarding the CS tests, CSV-1000 generated more expected information gain than Pelli–Robson, and the qCSF test with 25 trials generated more expected information gain than CSV-1000, whether scored with AULCSF or with CSF at six spatial frequencies. The active learning-based qVA and qCSF tests have the potential to generate more expected information gain than traditional paper chart tests. Although we have specifically applied it to compare VA and CS tests, expected information gain is a general concept that can be used to compare measurements in any domain.

## Introduction

Measurement serves as the bedrock of the scientific method and finds application across a diverse range of disciplines, encompassing physics, biology, engineering, social science, and medicine^1–4^. It provides the empirical data essential for hypotheses testing, theory formulation, performance evaluation, and diagnostic procedures^5–9^. Despite the development of numerous quality metrics designed to assess individual measurements and the concordance between two unidimensional measurements^10–12^, the task of comparing innovative measurements to the established gold standards remains a formidable challenge. This challenge is particularly pronounced when the novel measurements yield outcomes of distinct or greater dimensionality. Furthermore, conventional metrics, such as those gauging test–retest variability, primarily quantify the uncertainties associated with measurement outcomes and do not encompass an evaluation of the knowledge accrued through the act of measurement.

Viewed through the lens of information theory^13–15^, measurement is conceptually defined as “a set of observations that reduce uncertainty, with the result expressed as a quantity”^16^. In order to precisely quantify the extent of new knowledge derived from measurement, we advocate for the application of expected information gain, also referred to as expected mutual information^17^. While the concept of information gain^18^ has found extensive use in machine learning, where it serves as a fundamental criterion for optimizing decision trees^19^ and guides the selection of optimal stimuli in active learning algorithms^20–22^, its applications have largely centered on assessing relative information gain among different features or stimuli in the learning process. However, in this particular investigation, we employ it to quantitatively assess the amount of information acquired through measurements conducted on a population.

### Computing the expected information gain

Two equivalent methodologies exist for calculating the expected information gain. The first approach hinges on the reduction of uncertainty regarding the property being measured, often referred to as the “truth”, following the measurement. In contrast, the second approach is grounded in the disparity between the uncertainty associated with all conceivable measurement outcomes within a population and the projected remaining uncertainty after the execution of a measurement. While the first approach is a direct derivation from the concept of expected information gain, the second approach, in practice, tends to be more straightforward to implement. Both approaches are presented in the following section, and additional verification of their equivalence can be located in Appendix 1.

**Figure 1:**
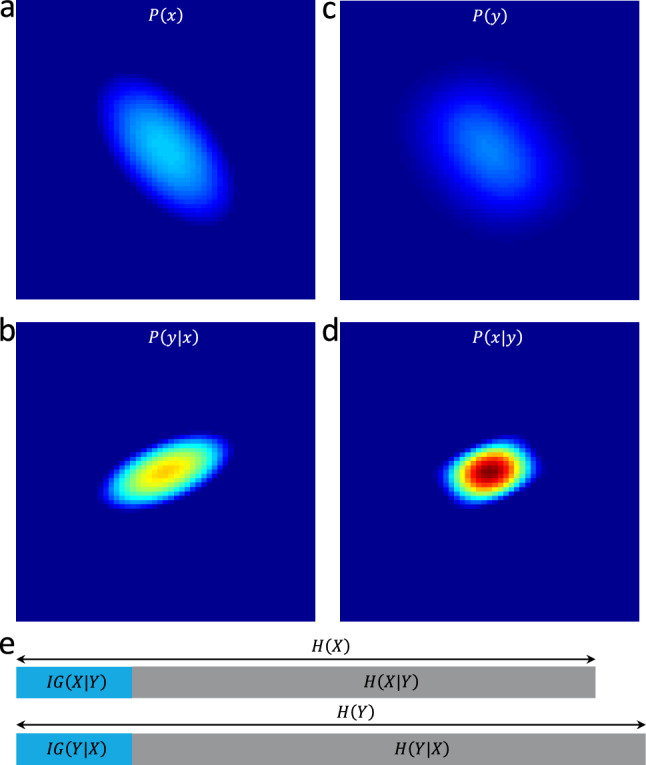
(**a**) Probability distribution of a quantity $x$ occurring in population $X, P\left(x\right)$. (**b**) Probability distribution of obtaining measurement $y$ given $x$ is the true value being measured: $P\left(y|x\right)$. (**c**) Probability distribution of obtaining a measurement $y$ from all potential measurement outcomes $Y$ regardless of the underlying true value, $P(y)$. (**d**) Posterior distribution of $X, P\left(x|y\right),$ following a measurement outcome $y$. (**e**) Expected information gain $IG(X|Y)$ is the difference between $H\left(X\right)$ and the expected posterior entropy $H\left(X|Y\right)$. Expected information gain $IG\left(Y|X\right)$ is the difference between $H\left(Y\right)$ and the expected residual entropy $H\left(Y|X\right)$. $IG\left(X|Y\right)=IG(Y|X)$.

The first approach to calculate expected information gain begins by considering the property to be measured, referred to as the “truth”. This property is represented by a random variable $X$, with a probability density function denoted as $P(x)$ (Fig. 1a). We also consider all possible outcomes of the measurement, represented by a random variable $Y$, with its own probability density function $P(y)$ (Fig. 1c). Following each measurement, the outcome is represented by a probability distribution $P(y|x)$ (Fig. 1b). By applying Bayes’ rule, we can derive the posterior distribution of $x$, denoted as $P(x|y)$(Fig. 1d), using the formula: $P\left(x|y\right)=P\left(y|x\right)P(x)/P(y)$, where $P\left(y\right)={\int }_{X}P\left(y|x\right)P\left(x\right)dx$. Shannon entropy is then employed to quantify the level of uncertainty associated with $X$ before any measurement:

1 $$H\left(X\right)=-\int\limits_{X}P\left(x\right){\log}_{2}\left(P\left(x\right)\right)dx.$$

Following a measurement, the expected entropy of $X$ is determined as:

2 $$H\left(X|Y\right)=-\int\limits_{Y}\left(\int\limits_{X}P\left(x|y\right){\log}_{2}\left(P\left(x|y\right)\right)dx\right)P\left(y\right)dy,$$

and subsequently, the expected information gain is computed (Fig. 1e):

3 $$IG\left(X|Y\right)=H\left(X\right)-H\left(X|Y\right)=-\int\limits_{X}P\left(x\right){\text{log}}_{2}\left(P\left(x\right)\right)dx+\int\limits_{Y}\left(\int\limits_{X}P\left(x|y\right){\text{log}}_{2}\left(P\left(x|y\right)\right)dx\right)P\left(y\right)dy.$$

This approach quantifies the reduction in uncertainty regarding the property X as a result of the measurement outcomes represented by $Y.$

The second approach to compute expected information gain also begins by considering probability density functions: $P\left(x\right),$ $P\left(y\right)$ and $P(y|x)$ (Fig. 1a–c). Using Shannon entropy, we first assess the level of uncertainty associated with Y based on the distribution of all possible measurement outcomes:

4 $$H\left(Y\right)=-\int\limits_{Y}P\left(y\right){\log}_{2}\left(P\left(y\right)\right)dy.$$

Next, we determine the expected residual uncertainty of Y after a measurement:

5 $$H\left(Y|X\right)=-\int\limits_{X}\left(\int\limits_{Y}P\left(y|x\right){\log}_{2}\left(P\left(y|x\right)\right)dy\right)P\left(x\right)dx.$$

Finally, the expected information gain (Fig. 1e) is computed as:

6 $$IG\left(Y|X\right)=H\left(Y\right)-H\left(Y|X\right)=-\int\limits_{Y}P\left(y\right){\log}_{2}\left(P\left(y\right)\right)dy+\int\limits_{X}\left(\int\limits_{Y}P\left(y|x\right){\log}_{2}\left(P\left(y|x\right)\right)dy\right)P\left(x\right)dx.$$

This second approach assesses how much uncertainty remains in $Y$ after obtaining the measurement outcomes represented by $P(y|x)$. In essence, it quantifies the reduction in uncertainty associated with Y as a result of measuring $X$. Both approaches yield equivalent results and provide valuable insights into the information gained through measurements in different ways.

### A practical illustration of expected information gain

**Figure 2:**
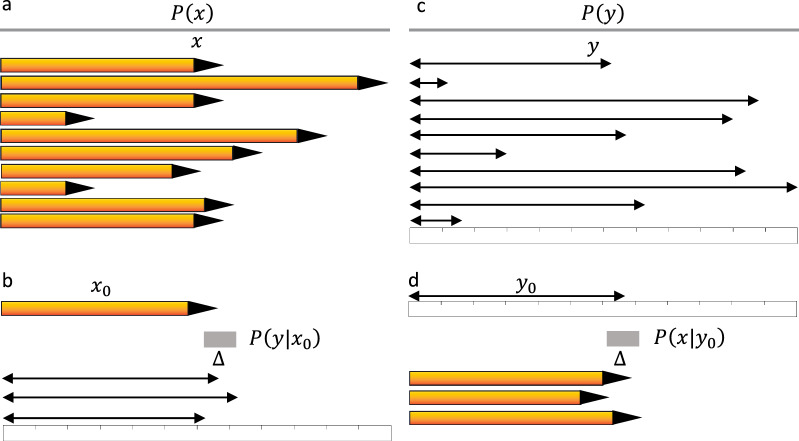
An illustration of expected information gain from measurement of the length of pencils using a ruler with unit $\Delta$. (**a**) A uniformly distributed pencil length $X$, $P\left(x\right)$. (**b**) Outcome distribution from measuring the length of a pencil with true length ${x}_{0}$, P $\left(y|{x}_{0}\right)$. (**c**) A uniformly distributed outcomes $Y$, $P\left(y\right).$ (**d**) Posterior distribution of $x$ based on measurement outcome ${y}_{0}, P(x|{y}_{0}).$

We present a practical illustration of expected information gain using two rulers, considering both the first (Fig. 2a,d) and second (Fig. 2b,c) approaches:

- *First approach* For a ruler with a unit $\Delta$, the probability distribution of measuring an object with length $x$ is represented by $P(y|x) = U(x-\Delta /2, x+\Delta /2)$, where $U(m, n)$ is a uniform distribution with boundaries $m$ and $n$. If this ruler is used to measure objects with lengths between $0$ and $L$ with equal probability, the probability distribution for object lengths is $P(x) = U(0, L)$. The entropy of X before any measurement $H(X)$ is:
  7a $$H\left(X\right)=-\int\limits_{0}^{L}U\left(0,L\right){\text{log}}_{2}\left(U\left(0,L\right)\right)dx={\text{log}}_{2}\left(L\right).$$
  The outcome distribution P(y) can also be determined:
  7b $$P\left(y\right)=\int\limits_{0}^{L}U\left(x-\frac{\Delta }{2}, x+\frac{\Delta }{2}\right)U\left(0,L\right)dx=U\left(0,L\right).$$
  The entropy of $X$ after considering $Y$ is:
  7c $$H\left(X|Y\right)=-\int\limits_{0}^{L}\int\limits_{0}^{L}U\left(x-\frac{\Delta }{2}, x+\frac{\Delta }{2}\right){\text{log}}_{2}\left(U\left(x-\frac{\Delta }{2}, x+\frac{\Delta }{2}\right)\right)dxU\left(0,L\right)dy={\text{log}}_{2}\left(\Delta \right).$$
  The expected information gain is then computed as:
  7d $$IG\left(X|Y\right)=H\left(X\right)-H\left(X|Y\right)={\text{log}}_{2}(L/\Delta ).$$
- *Second approach* The entropy of Y (H(Y)) is calculated:
  7e $$H\left(Y\right)=-\int\limits_{0}^{L}U\left(0,L\right){\text{log}}_{2}\left(U\left(0,L\right)\right)dy={\text{log}}_{2}\left(L\right).$$
  The expected residual uncertainty of $Y$ after considering $X$ is determined:
  7f $$H\left(Y|X\right)=-\int\limits_{0}^{L}\int\limits_{0}^{L}U\left(x-\frac{\Delta }{2}, x+\frac{\Delta }{2}\right){\text{log}}_{2}\left(U\left(x-\frac{\Delta }{2}, x+\frac{\Delta }{2}\right)\right)dyU\left(0,L\right)dx={\text{log}}_{2}\left(\Delta \right).$$
  The expected information gain is then computed as:
  7g $$IG\left(Y|X\right)=H\left(Y\right)-H\left(Y|X\right)={\text{log}}_{2}(L/\Delta ).$$

Consider two one-foot-long rulers, one with a one-inch unit and the other with a 1/16-inch unit. Using both approaches, the expected information gains are calculated as ${\text{log}}_{2}(12/1))$= 3.58 bits and ${\text{log}}_{2}(12/(1/16)) = 7.58$ bits for the two rulers, respectively. These values correspond to 12 and 192 distinct length classes, consistent with the number of units on the rulers. These calculations demonstrate how expected information gain can be applied to assess the knowledge gained from measurements using different rulers with varying units of measurement. It provides a quantitative measure of the information acquired through these measurements, which can be valuable in various contexts.

### Expected information gain for a unidimensional measurement with a normal outcome distribution

We next show how to derive the expected information gain for a unidimensional measurement with a normal outcome distribution and provide an intuitive interpretation. In this case, the probability distribution for measuring $y$ given $x$ is defined as $P(y|x) =\frac{1}{\sqrt{2\pi }\sigma }{e}^{-\frac{{(y-\mu (x))}^{2}}{2{\sigma }^{2}}},$ where $\mu (x)$ is the expected value of $x$. If both $x$ and $y$ are uniformly distributed in an interval of length $L$, then $H\left(X\right)={\text{log}}_{2}\left(L\right),$ $H\left(X|Y\right)={\text{log}}_{2}\left(\sigma \sqrt{2\pi e}\right)$, $H\left(Y\right)={\text{log}}_{2}\left(L\right),$ $H\left(Y|X\right)={\text{log}}_{2}\left(\sigma \sqrt{2\pi e}\right)$, and $IG\left(X|Y\right)=IG\left(Y|X\right)={\text{log}}_{2}\left(\frac{L}{4.13\sigma }\right).$ Intuitively, these values indicate the following: (1) $L/(4.13\sigma )$ classes: This represents the number of distinct classes or intervals into which the measurement outcomes can be categorized. Each class has a size of approximately $4.13\sigma$, which corresponds to the 98% confidence interval of the outcome distribution. In other words, this is a way to quantitatively express the granularity or resolution of the measurement. (2) The 98% Confidence Interval: The size of each class, 4.13σ, represents the range within which an observed measurement is likely to fall with a high level of confidence (98% confidence interval). This interval provides a measure of uncertainty associated with the measurement outcome.

### Overview of the current study

Expected information gain, which quantifies the knowledge gained through measurement, is not limited to unidimensional examples. It can be effectively used to compare measurements with any level of dimensionality. In this study, we extended its application to the assessment of visual acuity (VA) and contrast sensitivity (CS) tests, which inherently involve measurements with different dimensionalities. By applying expected information gain to VA and CS tests, we aimed to provide a quantitative basis for comparing these tests, considering their varying optotypes, outcome dimensionalities, and their ability to provide valuable knowledge.

Visual acuity is a crucial measure of visual function and is widely used for diagnosing and managing visual diseases, evaluating the effectiveness of treatments, and establishing professional standards^23–25^. The gold standard test, the ETDRS chart^26^, consisting of rows of five equal-sized optotypes, is used to generate a unidimensional VA threshold score for each patient. However, a newer test called the qVA test^27,28^ is introduced, which involves three equal-sized optotypes in each trial and generates a two-dimensional score, incorporating VA threshold and VA range. Importantly, this added dimension provides a more comprehensive assessment of visual acuity, and not considering it could lead to an incomplete evaluation^28^.

The contrast sensitivity function is an increasingly important measure in clinical research and clinical trials as it offers a more comprehensive characterization of spatial vision compared to VA^29,30^. Multiple instruments have been developed to measure CS^20,31,32^, each using different optotypes and producing scores with varying dimensionalities. For example, the Pelli–Robson test^32^ uses unfiltered Sloan letter stimuli and generates a unidimensional CS score at one spatial frequency, while the CSV-1000 test^33^ uses windowed sinewave grating stimuli and provides a four-dimensional CS score at four spatial frequencies. Another test, the qCSF^20,34^, uses filtered Sloan letter stimuli and generates both a unidimensional area under the log CSF (AULCSF) score and a six-dimensional CS score at six spatial frequencies. This diversity in CS tests and their outcome dimensions poses challenges when comparing their effectiveness.

While many studies have assessed the accuracy, test–retest variability, sensitivity, and specificity of VA and CS tests^28,35–47^, comparing them has proven difficult due to differences in optotypes, optotype arrangements, and outcome dimensionalities. Existing metrics often focus solely on the uncertainty of measurement outcomes and do not quantify the knowledge gained through these measurements. This study aimed to address these challenges by employing the concept of expected information gain to compare VA and CS tests. Computer simulations were used to calculate and compare the expected information gain and the number of classes derived from the Snellen chart^48^, ETDRS chart^26^, and qVA test^27^ for VA assessment, as well as the Pelli–Robson chart^32^, CSV-1000 chart (Vector Vision, Houston, Texas)^33^, and qCSF test^20,34^ in populations with uniform distributions, as well as populations with distributions based on experimental data. This approach provides a quantitative and knowledge-based method for comparing these essential vision tests.

## Methods

### Apparatus

All the simulations and analyses were conducted on a Dell computer with Intel Xeon W-2145 @ 3.70 GHz CPU (8 cores and 16 threads) and 64 GB installed memory (RAM) with Matlab R2019a (MathWorks Corp., Natick, MA, USA) and R (R Core Team, 2020).

### Visual acuity tests

#### Simulated observers

**Figure 3:**
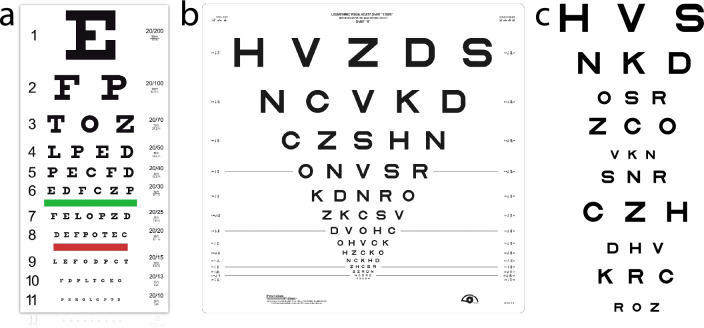
(**a**) A Snellen chart (Image courtesy of Precision Vision, Inc). (**b**) An ETDRS chart (Image courtesy of Precision Vision, Inc). (**c**) A subset of potential stimuli in qVA.

We conducted two simulations using the Snellen, ETDRS, and qVA tests (Fig. 3). In Simulation 1, we simulated 1386 observers from a uniform distribution of VA threshold $\left({\theta }_{Threshold}^{VA}\right)$ and VA range $\left({\theta }_{Range}^{VA}\right)$, with ${\theta }_{Threshold}^{VA}\in \left[-0.3, 1.0\right]$ logMAR and sampled every 0.02 logMAR, and ${\text{log}}_{10}({\theta }_{Range}^{VA})$ $\in \left[-1.0, 0\right]$ and sampled every 0.05 ${\text{log}}_{10}$ units. In Simulation 2, we simulated 1386 observers from the population distribution of VA threshold and VA range derived from an existing qVA dataset of 14 eyes tested with Bangerter foils^28,49^. The original experiment was conducted at the Ohio State University. Written consent was obtained from all the participants before the experiment. The study protocol was approved by the institutional review board of human subject research of The Ohio State University and adhered to the tenets of the Declaration of Helsinki.

#### Visual acuity behavioral function

For each simulated observer, the discriminability ($d^{\prime}$) for an optotype of size $s$ is described by the VA behavioral function (Patent No. US 10758120B2)^27^:

8 $${{\mathrm{log}}_{10}(d}^{\mathrm{^{\prime}}}(s|{\theta }^{VA}))={\mathrm{log}}_{10}\left(6\right)+\frac{\omega }{2{\theta }_{Range}^{VA}}\left(s-{\theta }_{Threshold}^{VA}\right)-\frac{1}{2}{\mathrm{log}}_{10}\left(8+{10}^{\frac{\omega }{{\theta }_{Range}^{VA}}\left(s-{\theta }_{Threshold}^{VA}\right)}\right),$$

where ${\theta }^{VA}=({\theta }_{Threshold}^{VA},{\theta }_{Range}^{VA})$, ${\theta }_{Threshold}^{VA}$ is VA threshold, corresponding to the optotype size at ${d}^{\mathrm{^{\prime}}}$ = 2, ${\theta }_{Range}^{VA}$ is the VA range of the behavioral function, that is, the range of optotype sizes that covers ${d}^{\mathrm{^{\prime}}}$=1 to ${d}^{\mathrm{^{\prime}}}=$ 4 performance levels, and $\omega ={\mathrm{log}}_{10}35-{\mathrm{log}}_{10}1.25$.

In these VA tests, observers identify optotypes from the 10 Sloan letters. We can compute the probability of obtaining $m$ correct responses in the 10-alternative forced identification task in a row of $M$ randomly sampled optotypes from their *d*ʹ function (Appendix 2):

9 $$\mathrm{p}\left(m|s,M, {\theta }^{VA}\right)=f\left(m,M,{d}^{\mathrm{^{\prime}}}\left(s|{\theta }^{VA}\right)\right),$$

where $f\left(.\right)$ is derived from signal detection theory by considering chart design, i.e., the number of optotypes in a row and whether optotypes in each row are sampled from the 10 Sloan letters with or without replacement.

#### Snellen chart

The Snellen chart (Fig. 3a) has 11-rows, with 1, 2, 3, 4, 5, 6, 7, 8, 8, 8, and 9 optotypes in each row and optotype size descending from 1.0 to − 0.3 logMAR. Each simulated observer was tested with the standard procedure^50^. The probability of correctly identifying $m$ optotypes in a row is determined by Eq. (9) with varying $M$ across rows. Starting from the top row, the observer must correctly identify at least half of the optotypes on a row before proceeding to the next row. If they can’t identify the optotype on the top row, the VA score was 1.1 logMAR; otherwise, the VA score is equal to the size of the optotypes in the last row with at least 50% correct identification. The VA score could therefore take 12 potential values. For each simulated observer ${x}_{i}$, we repeated the test 1000 times to obtain the distribution of test scores $P\left({y}_{j}|{x}_{i}\right),$ where $j=1, \dots , 12.$

#### ETDRS chart

The ETDRS chart (Fig. 3b) has 14 five-optotype rows, with optotype size descending from 1.0 to − 0.3 logMAR. Each simulated observer was tested with the standard procedure^26,50^. The probability of correctly identifying $m$ optotypes in a row is determined by Eq. (9) with $M=5$. Four different termination rules were simulated. Starting from the top row, the test could stop after the observer makes three, four, or five mistakes in identifying the optotypes in a row or continue until the observer is tested with the entire chart. The VA score is computed as 1.1–0.02*n*, where $n$ is the number of correctly identified optotypes, with 71 potential values. For each simulated observer ${x}_{i}$, we repeated the test 1000 times to obtain the distribution of test scores $P\left({y}_{j}|{x}_{i}\right),$ where $j=1, \dots , 71.$

#### qVA test

The qVA (Fig. 3c) is a Bayesian active learning visual acuity test^27^. Its stimulus space consists of optotypes of 91 linearly spaced sizes from − 0.5 to 1.3 logMAR, with a 0.02 logMAR resolution. Starting with a weak prior distribution of VA threshold and VA range in a two-dimensional space that has 700 linearly spaced VA thresholds (between − 0.5 and 1.3 logMAR) and 699 log-linearly spaced VA ranges (between 0.1 and 1.5 logMAR), it uses an active learning procedure to test the observer with the optimal stimulus in each trial and generates the posterior distribution of VA threshold and range. Each simulated observer was tested with 5, 15, or 30 rows (corresponding to 15, 45, or 90 optotypes). The probability of correctly identifying $m$ optotypes in a row is determined by Eq. (9) with $M=3$. We computed the mean VA threshold and range from their posterior distributions in each test and quantized them into 86 and 56 discrete scores with a 0.02 logMAR resolution, with a total of 4816 potential combinations. For each simulated observer ${x}_{i}$, we repeated the test 1000 times to obtain the two-dimensional distribution of test scores $P\left({y}_{j}|{x}_{i}\right),$ with $j=1, \dots ,4816$. We also computed the distribution of VA threshold $P\left({y}_{Threshold,j}|{x}_{i}\right)$ by marginalizing P $\left({y}_{j}|{x}_{i}\right)$:

10 $$P\left({y}_{Threshold,j}|{x}_{i}\right)=\sum_{range}P\left({y}_{j}|{x}_{i}\right),$$

where *j* = 1,…,86.

#### Information gain

We first computed $\mathrm{P}\left({y}_{j}\right)$ from $P\left({y}_{i}|{x}_{i}\right)$ for each test:

11 $$P\left({y}_{j}\right)=\frac{1}{I}\sum_{i=1}^{I}P({y}_{j}|{x}_{i}),$$

where *I* = 1386 in both simulations. We then computed the total entropy of each test of a population:

12 $$H\left(Y\right)=-\sum_{j=1}^{J}P({y}_{j}){\text{log}}_{2}(P({y}_{j})),$$

where *J* = 12, 71, 86, 4816 for the Snellen, ETDRS, VA threshold from qVA, and VA threshold and VA range from qVA. The expected residual entropy was computed as:

13 $$\boldsymbol{ }H\left(Y|X\right)=- \frac{1}{I}\sum_{i=1}^{I}\sum_{j=1}^{J}P({y}_{j}|{x}_{i}){\text{log}}_{2}(P({y}_{j}|{x}_{i})),$$

and, finally expected information gain can be obtained:

14 $$IG\left(Y|X\right)=H\left(Y\right)- H\left(Y|X\right).$$

### Contrast sensitivity tests

#### Simulated observers

**Figure 4:**
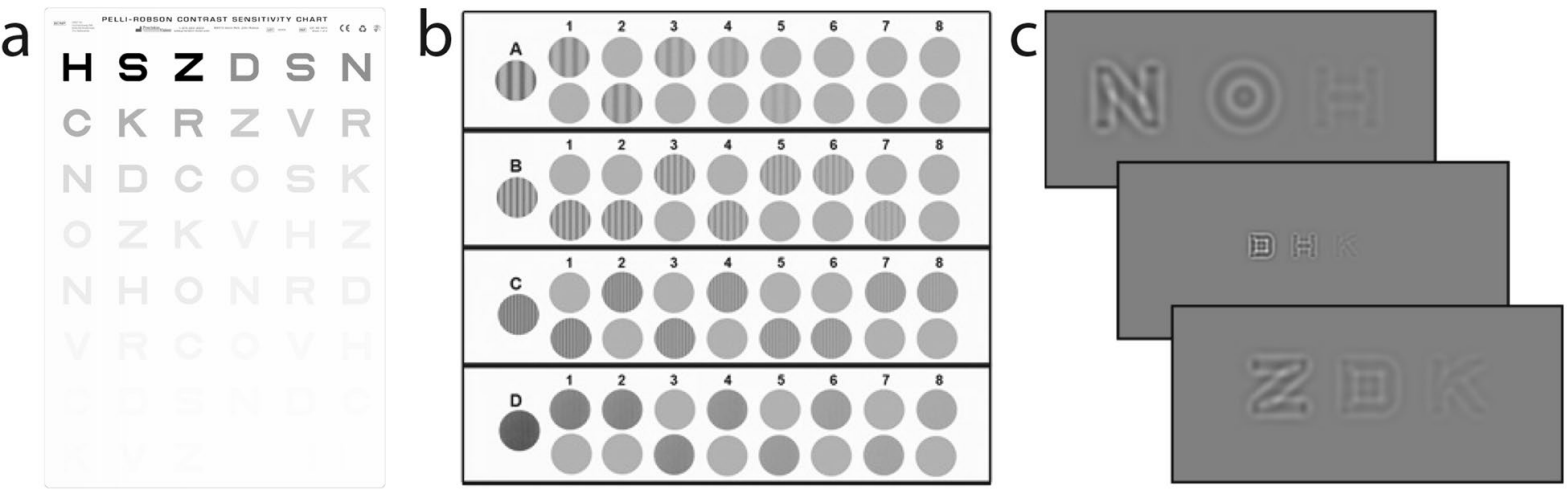
(**a**) A Pelli–Robson chart (Image courtesy of Precision Vision, Inc.). (**b**) A CSV-1000 chart (Reproduced with permission from GUARDiON Health Sciences, Inc.). (**c**) A subset of potential stimuli in qCSF.

We conducted two simulations using the Pelli–Robson chart, CSV-1000 chart, and qCSF test (Fig. 4). In Simulation 1, we simulated 1911 observers from a uniform distribution of peak gain $\left({\theta }_{PG}^{CSF}\right),$ peak spatial frequency $\left({\theta }_{PF}^{CSF}\right)$, and band width $\left({\theta }_{BH}^{CSF}\right)$, with ${{\text{log}}_{10}(\theta }_{PG}^{CSF})\in \left[0.3, 2.3\right]$ sampled every 0.10 log_10_ units, ${{\text{log}}_{10}(\theta }_{PF}^{CSF})$ $\in \left[-0.3, 0.9\right]$ sampled every 0.10 log_10_ units, and ${{\text{log}}_{10}(\theta }_{BH}^{CSF})$ $\in \left[0.3, 0.6\right]$ sampled every 0.05 log_10_ units. In Simulation 2, we simulated 1911 observers from the population distribution of CSF parameters derived from two existing qCSF datasets, one consisted of 112 eyes tested in three luminance conditions^38^ and the other of 14 eyes tested with Bangerter foils^28^, using a hierarchical Bayesian model^49,51^. The original experiments were conducted at the Ohio State University. Written consent was obtained from all the participants before the experiment. The study protocol was approved by the institutional review board of human subject research of The Ohio State University and adhered to the tenets of the Declaration of Helsinki.

#### Letter and grating contrast sensitivity functions

The letter CSF, which specifies contrast sensitivity ${S}_{letter}\left(f\right)$ for filtered letters of different sizes at center spatial frequency $f$, can be described with a log parabola function with three parameters ${\theta }^{CSF}=\left({\theta }_{PG}^{CSF},{\theta }_{PF}^{CSF},{\theta }_{BH}^{CSF}\right)$^20,52,53^:

15 $${\mathrm{log}}_{10}\left({S}_{letter}\left(f|{\theta }^{CSF}\right)\right)= {\mathrm{log}}_{10}\left({\theta }_{PG}^{CSF}\right)-\frac{4}{{\mathrm{log}}_{10}\left(2\right)}{\left(\frac{{\mathrm{log}}_{10}\left(f\right)-{\mathrm{log}}_{10}\left({\theta }_{PF}^{CSF}\right)}{{\theta }_{BH}^{CSF}}\right)}^{2},$$

where ${\theta }_{PG}^{CSF}$ is the peak gain, ${\theta }_{PF}^{CSF}$ is the peak spatial frequency (cycles/degree), and ${\theta }_{BH}^{CSF}$ is the bandwidth (octaves) at half-height. For an observer with peak gain ${\theta }_{PG}^{CSF}$, peak spatial frequency ${\theta }_{PF}^{CSF},$ and bandwidth ${\theta }_{BH}^{CSF}$, the probability of correct identification of a bandpass-filtered optotype with contrast $c$ and center spatial frequency $f$ is described with a Weibull psychometric function^34^:

16 $$p\left(correct|{\theta }^{CSF},f,c \right)=g+\left(1-g-\frac{\lambda }{2}\right)[1-\mathrm{exp}\left[- {\left(c\times {S}_{letter}\left(f|{\theta }^{CSF}\right)\right)}^{b}\right]],$$

where $g$ is the guessing rate, $\lambda$ = 0.04 is the lapse rate, and $b$ determines the steepness of the psychometric function. Because they both use a 10-alternative forced identification task, $g=0.1$ and $b=4.05$ for the Pelli–Robson chart and qCSF test.

The grating CSF, which specifies contrast sensitivity ${S}_{grating}\left(f\right)$ for gratings of the same size at spatial frequency $f$, needs to be corrected for the increased number of cycles with increasing spatial frequency^54^. For the grating stimuli used in the CSV-1000:

17 $${\mathrm{log}}_{10}\left({S}_{grating}\left(f|{\theta }^{CSF}\right)\right)= {\mathrm{log}}_{10}\left({\theta }_{PG}^{CSF}\right)-\frac{4}{{\mathrm{log}}_{10}\left(2\right)}{\left(\frac{{\mathrm{log}}_{10}\left(f\right)-{\mathrm{log}}_{10}\left({\theta }_{PF}^{CSF}\right)}{{\theta }_{BH}^{CSF}}\right)}^{2}+{\mathrm{log}}_{10}\left(0.539f\right).$$

For the yes/no task in the first column of the CSV-1000 test, we used a high-threshold model. That is, the simulated observer says yes if the stimulus contrast > threshold (= 1/${S}_{grating}\left(f|{\theta }^{CSF}\right)).$ The simulated observer says no otherwise. For the two-alternative forced choice task in CSV-1000, we replace ${S}_{letter}\left(f|{\theta }^{CSF}\right)$ with ${S}_{grating}\left(f|{\theta }^{CSF}\right)$ and set $g=0.5$, $b=3.06$ in Eq. (16) to compute the probability of making a correct response.

#### Pelli–Robson chart

The Pelli–Robson chart (Fig. 4a) consists of 16 optotype triplets of the same size and log-linearly spaced contrast between 0.56 and 100%^33^. At a viewing distance of 3 m, the center frequency of the optotypes is 3 c/d. The probability of correctly identifying each optotype in the chart is determined by Eq. (16). Starting from the top row, the test proceeds to the next triplets only if the observer correctly identifies at least two of the three optotypes in the current triplet. The CS of the observer is determined by the lowest contrast ${c}_{lowest}$ at which they correctly identify at least two of the three letters in the triplet: ${S}_{letter}\left(3c/d\right)=-{\text{log}}_{10}({c}_{lowest}$), with 17 potential contrast sensitivity scores. For each simulated observer ${x}_{i}$, we repeated the test 1000 times to obtain the distribution of test scores $P\left({y}_{j}|{x}_{i}\right),$ where $j=1, \dots , 17.$

#### CSV-1000 chart

The CSV-1000 chart (Fig. 4b) consists of CS tests at four spatial frequencies: 3, 6, 12, and 18 cycles/degree. Each test consists of 17 stimuli arranged in nine columns, with a single high-contrast vertical sinewave grating in the first column, and two test patches in the remaining eight columns, of which only one contains a vertical sinewave grating. The gratings are arranged with decreasing contrast from left to right, with contrast from − 0.70 to − 2.08, − 0.91 to − 2.29, − 0.61 to − 1.99, and − 0.17 to − 1.55 log10 units in the four rows. Going through all four rows starting from the top, the observer is first required to perform a yes/no task on the first column in each row. If the observer can’t see the stimulus in the first column, the test stops for that row and the observer’s contrast sensitivity is:

18 $$S\left(f\right)=\left\{\begin{array}{l}-{\text{log}}_{10}\left({c}_{first column}\left(f\right)\right)-0.3, f=3, 6, 12\\ 0.01, f=18\end{array}\right..$$

If the observer can see the stimulus in the first column, they proceed to identify the location of the patch that contained the grating in each column with a three-alternative forced choice response: top, bottom, or blank. We treat blank as an incorrect response. The lowest contrast at which the observer correctly identifies the location of the grating is used to determine CS in the test: $S\left(f\right)=-{\text{log}}_{10}({c}_{lowest}(f)$). The result is a four-dimensional CS score sampled at four spatial frequencies. Because there are 10 potential CS scores in each spatial frequency, there are therefore a total of ${10}^{4}$ potential CS functions. For each simulated observer ${x}_{i}$, we repeated the test 1000 times to obtain the distribution of test scores $P\left({y}_{j}|{x}_{i}\right),$ where $j=1, \dots , {10}^{4}.$

#### qCSF test

The qCSF (Fig. 4c) is a Bayesian active learning contrast sensitivity test^20,34^. Its stimulus space consists of 128 log-linearly spaced contrasts (from 0.002 to 1.0) and 19 log-linearly spaced spatial frequencies (from 1.19 to 30.95 c/d). Although a four-parameter truncated log parabola has been used in the qCSF test^20^, we removed the truncation parameter in the simulations because we didn’t score the simulated observers in very low spatial frequencies. Starting with a weak prior distribution of peak gain, peak frequency and bandwidth in a three-dimensional space that has 60 log-linearly spaced peak gains (from 1.05 to 1050), 40 log-linearly spaced peak frequencies (from 0.1 to 20 c/d), and 27 log-linearly spaced bandwidth (from 1 to 9 octaves), it uses an active learning procedure to test the observer with the optimal stimuli in each trial and computes the posterior distribution of the three CSF parameters in the afore-mentioned three-dimensional space. In each trial, three filtered optotypes with the same center spatial frequency but four, two, and one times the optimal contrast (capped at 0.9) are presented. The observer could be tested with 15, 25 and 50 trials. For each simulated observer ${x}_{i}$, we repeated the test 1000 times to obtain distributions of the unidimensional AULCSF $P\left({y}_{AULCSF,j}|{x}_{i}\right)$ and the six-dimensional CSF score $P\left({y}_{CSF,j}|{x}_{i}\right)$ at six spatial frequencies (1, 1.5, 3, 6, 12 and 18 c/d). Sampling the scores at 0.05 log10 resolution, *j* = 1, …, 57 for ${y}_{AULCSF,j}$, and *j* = 1,…, 253,492 for ${y}_{CSF,j}$.

#### Information gain

Equations (11)–(14) were used to compute $\mathrm{P}\left({y}_{j}\right), H\left(Y\right)$, $H\left(Y|X\right),$ and $IG\left(Y|X\right)$, with *I* = 1911 for the two simulations, and *J* = 17, 10,000, 57, 253,492 for the Pelli–Robson, CSV-1000, AULCSF from qCSF, and CSF from qCSF.

## Results

### Visual acuity tests

**Figure 5:**
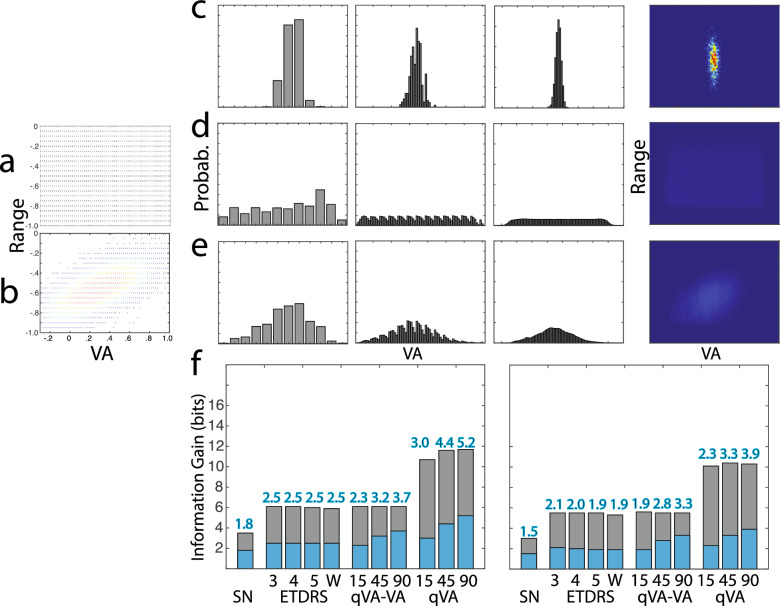
Distributions and information gain in visual acuity tests. (**a,b**) Distributions of VA threshold and VA range of the simulated observers in Simulations 1 and 2. (**c**) From left to right: Distributions of the test scores $P\left({y}_{j}|{x}_{i}\right)$ of one representative simulated observer ${x}_{i}$ in the Snellen, ETDRS (3-mistake rule), and qVA test (45 optotypes), with results from the qVA test scored as VA-alone, and as both VA threshold and VA range. (**d,e**) Distributions of the test scores $P\left({y}_{j}\right)$ of the populations in Simulations 1 and 2, respectively. (**f**) Expected information gain $IG\left(Y|X\right)$ of the various tests in Simulation 1 (left) and Simulation 2 (right). For ETDRS, results from the 3-, 4-, 5-mistake and whole chart rules are shown. For qVA, results from testing with 15, 45, and 90 optotypes are shown. The blue bars represent $IG\left(Y|X\right)$; the gray bars represent residual entropy $H\left(Y|X\right)$.

The VA threshold and VA range distributions of the observers in the two simulations are shown in Fig. 5a,b. Figure 5c shows distributions of the test scores $P\left({y}_{j}|{x}_{i}\right)$ of one representative simulated observer ${x}_{i}$ in the Snellen, ETDRS (3-mistake rule), and qVA test, with results from the qVA test scored as VA-alone, and as both VA threshold and VA range. Figure 5d,e show the distributions of the test scores $P\left({y}_{j}\right)$ of the populations in Simulations 1 and 2, respectively. Because a uniform $X$ distribution is used in Simulation 1, the corresponding $P\left({y}_{j}\right)^{\prime}s$ from the tests are nearly uniform. On the other hand, $P\left({y}_{j}\right)^{\prime}s$ in Simulation 2 are more concentrated because the population is more concentrated.

The total entropy $H\left(Y\right)$, the expected residual entropy $H\left(Y|X\right),$ the expected information gain $IG\left(Y|X\right)$, the expected number of classes $N\left(Y|X\right)$, the average number of optotypes tested, the expected information gain per optotype tested, and the ratio of expected information gain versus the ${log}_{2}$ of the number of optotypes tested from the three tests in the two simulations are listed in Table 1. The expected information gain $IG\left(Y|X\right)$ is also plotted in Fig. 5f. As expected, $H\left(Y\right)$ in Simulation 2 is less than the corresponding $H(Y)$ in Simulation 1 for all the tests because of the more concentrated $P\left({y}_{j}\right)$ resulted from a narrower range of simulated observers. As a result, $IG\left(Y|X\right)$ and $N\left(Y|X\right)$ in Simulation 2 are less than those in Simulation 1.

**Table 1:** Entropy, information gain and number of classes from visual acuity tests.

		Snellen	ETDRS				$q\mathrm{VA}$					
			# Mistakes			Whole chart	VA alone			VA + Range		
			3	4	5		15	45	90	15	45	90
Simulation 1	H(Y) (bits)	3.5	6.1	6.1	6.0	5.9	6.1	6.1	6.1	10.7	11.6	11.7
	H(Y|X) (bits)	1.6	3.5	3.5	3.6	3.4	3.8	2.9	2.4	7.8	7.2	6.5
	IG (bits)	1.8	2.5	2.5	2.5	2.5	2.3	3.2	3.7	3.0	4.4	5.2
	N(Y|X)	3.6	5.7	5.7	5.6	5.6	5.0	9.1	12.7	7.9	20.5	37.2
	# Optotypes	24.7	41.4	45.5	51.8	70	15	45	90	15	45	90
	IG/Optotype(bits)	0.07	0.06	0.06	0.05	0.04	0.16	0.07	0.04	0.20	0.10	0.06
	IG/log2(# Optotype)(bits)	0.40	0.47	0.46	0.44	0.40	0.60	0.58	0.56	0.76	0.79	0.80
Simulation 2	H(Y) (bits)	3.0	5.5	5.5	5.5	5.3	5.6	5.5	5.5	10.1	10.4	10.3
	H(Y|X) (bits)	1.6	3.5	3.5	3.6	3.4	3.7	2.8	2.3	7.7	7.2	6.5
	IG (bits)	1.5	2.1	2.0	1.9	1.9	1.9	2.8	3.3	2.3	3.3	3.9
	N(Y|X)	2.8	4.2	4.0	3.8	3.8	3.8	6.8	9.5	5.1	9.6	14.6
	# Optotypes	23.8	44.7	48.4	55.1	70	15	45	90	15	45	90
	IG/Optotype(bits)	0.06	0.05	0.04	0.03	0.03	0.13	0.06	0.04	0.16	0.07	0.04
	IG/log2(# Optotype)(bits)	0.32	0.38	0.36	0.33	0.32	0.49	0.50	0.50	0.60	0.59	0.60

To check the validity of the simulations, we also estimated expected information $IG\left(Y|X\right)$ of the Snellen and ETDRS tests from their reported test–retest variabilities (TRV = 1.96 $\sigma$), with the assumption that the outcome distributions are normal and have the same TRV for observers with different acuities. For both tests, the outcome scores cover − 0.3 to 1.0 logMAR, expected information gain can be computed with $IG\left(Y|X\right)$ = ${\mathrm{log}}_{2}\left(\frac{\mathrm{L}}{4.13\sigma }\right),$ where $L=1.3$ logMAR. For the Snellen chart, the typical TRV is 0.23 logMAR^35^, with $\sigma =\frac{TRV}{1.96}=0.117$ logMAR, and the expected information gain is 1.4 bits. For the ETDRS chart, the typical TRV is 0.11 logMAR^35^, with $\sigma =\frac{TRV}{1.96}=0.056$ logMAR, and the expected information gain is 2.5 bits. These estimated values are largely consistent with our results in Simulation 1.

For the qVA test, we also computed expected information gain from an existing dataset with 14 eyes tested in four Bangerter foil conditions^28^. In this dataset, VA threshold is between − 0.15 and 0.68 logMAR, and VA range is between 0.12 and 0.63 logMAR. We computed the total entropy, residual entropy and information gain based on the posterior distributions from single tests rather than distributions of test scores from repeated tests. In the qVA test, the posterior distributions from single tests are broader than those derived from repeated tests until about 45 optotypes are tested and converge to those from repeated tests afterwards^28^. The expected information gain based on VA threshold alone is 1.6, 2.6 and 2.9 bits after qVA test with 15, 45 and 90 optotypes, respectively, and the expected information gain based on VA threshold and range is 2.1, 3.2, and 3.6 bits after qVA test with 15, 45 and 90 optotypes, respectively. These results are largely consistent with those from Simulation 2.

In both simulations, ETDRS generated more expected information gain than Snellen. Scored with VA threshold alone or with both VA threshold and VA range, qVA with 15 rows (or 45 optotypes) generated more expected information gain than ETDRS. In terms of expected information gain per optotype tested, the qVA test with 45 optotypes scored with VA alone and with both VA and VA range was more efficient than the Snellen chart, which was in turn more efficient than the ETDRS chart. The different efficiencies were caused by different test designs and the distributions of acuity behavior used in the study. Interestingly, the ratio of expected information gain and the ${\text{log}}_{2}$ of the number of optotypes tested in qVA was essentially constant across test lengths and was greater than the corresponding scores in ETDRS and Snellen. The results suggest that the expected information gain increased linearly with the ${\text{log}}_{2}$ of the number of optotypes in the qVA test.

### Contrast sensitivity tests

**Figure 6:**
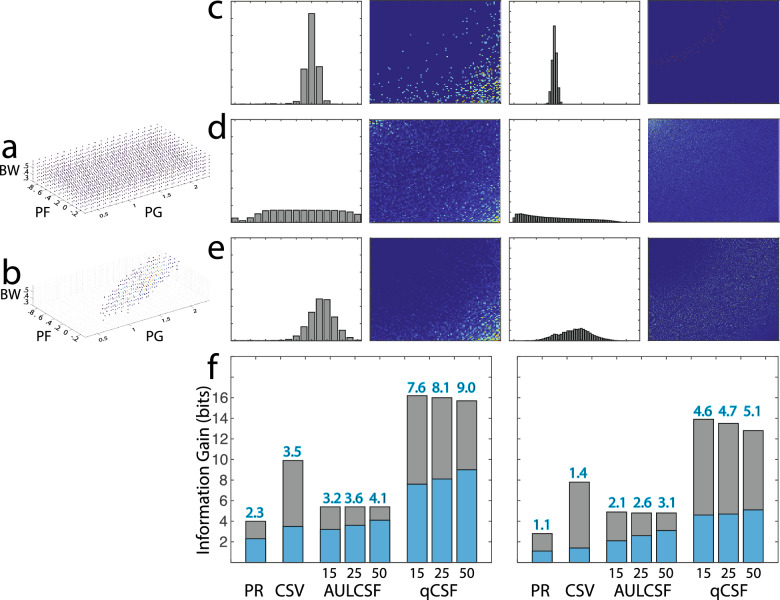
Distributions and information gain in contrast sensitivity tests. (**a,b**) Distributions of peak gain, peak spatial frequency, and bandwidth of the observers in Simulations 1 and 2. (**c**) From left to right: Distributions of the test scores $P\left({y}_{j}|{x}_{i}\right)$ of one representative simulated observer ${x}_{i}$ in the Pelli–Robson, CSV-1000, and qCSF test (25 trials), with results from the qCSF test scored as AULCSF, and CSF at six spatial frequencies. (**d,e**) Distributions of the test scores $P\left({y}_{j}\right)$ of the populations in Simulations 1 and 2, respectively. (**f**) Expected information gain $IG\left(Y|X\right)$ of the various tests in Simulation 1 (left) and Simulation 2 (right). For qCSF, results from testing with 15, 25, and 50 trials are shown. The blue bars represent $IG\left(Y|X\right)$; the gray bars represent residual entropy $H\left(Y|X\right)$.

The peak gain, peak spatial frequency, and bandwidth distributions of the observers in the two simulations are shown in Fig. 6a,b. Figure 6c shows distributions of the test scores $P\left({y}_{j}|{x}_{i}\right)$ of one representative simulated observer ${x}_{i}$ in the Pelli–Robson, CSV-1000, and qCSF test, with results from the qCSF test scored as AULCSF, and CSF at six spatial frequencies. Figure 6d,e show the distributions of the test scores $P\left({y}_{j}\right)$ of the populations $X$ in Simulations 1 and 2, respectively. Because a uniform $X$ distribution is used in Simulation 1, the corresponding $P\left({y}_{j}\right){\prime}s$ from the tests are more uniform. On the other hand, $P\left({y}_{j}\right){\prime}s$ in Simulation 2 are more concentrated because the observers are sampled in a narrower range.

The total entropy $H\left(Y\right)$, the expected residual entropy $H\left(Y|X\right),$ the expected information gain $IG\left(Y|X\right)$, the expected number of classes $N\left(Y|X\right),$ the average number of optotypes tested, the expected information gain per optotype tested, and the ratio of expected information gain versus the ${\text{log}}_{2}$ of the number of optotypes tested from the three tests in the two simulations are listed in Table 2. The expected information gain $IG\left(Y|X\right)$ is also plotted in Fig. 6f. As expected, $H\left(Y\right)$ in Simulation 2 is less than the corresponding $H(Y)$ in Simulation 1 in all the tests because of the more concentrated $P\left({y}_{j}\right)$ resulted from a narrower range of simulated observers. As a result, $IG\left(Y|X\right)$ and $N\left(Y|X\right)$ in Simulation 2 are less than those in Simulation 1.

**Table 2:** Entropy, information gain and number of categories from contrast sensitivity tests.

		Pelli–Robson	CSV-1000	$q\mathrm{CSF}$					
				AULCSF			CSF		
				15	25	50	15	25	50
Simulation 1	H(Y) (bits)	4.0	9.9	5.4	5.4	5.4	16.2	16.0	15.7
	H(Y|X) (bits)	1.7	6.4	2.2	1.8	1.4	8.5	7.8	6.7
	IG (bits)	2.3	3.5	3.2	3.6	4.1	7.6	8.1	9.0
	Categories	5.0	11.1	9.1	12.1	16.7	196.1	281.2	498.0
	# Optotypes	27.9	11.0	45	75	150	45	75	150
	IG/Optotype(bits)	0.08	0.32	0.07	0.05	0.03	0.17	0.11	0.06
	IG/log2(# Optotype)(bits)	0.48	1.01	0.58	0.58	0.56	1.39	1.31	1.24
Simulation 2	H(Y) (bits)	2.8	7.8	4.9	4.8	4.8	13.9	13.5	12.8
	H(Y|X) (bits)	1.7	6.5	2.7	2.3	1.7	9.4	8.8	7.8
	IG (bits)	1.1	1.4	2.1	2.6	3.1	4.6	4.7	5.1
	Categories	2.1	2.6	4.3	5.9	8.6	23.7	26.0	34.1
	# Optotypes	36.8	15.2	45	75	150	45	75	150
	IG/Optotype(bits)	0.03	0.09	0.05	0.03	0.02	0.10	0.06	0.03
	IG/log2(# Optotype)(bits)	0.21	0.35	0.38	0.41	0.43	0.83	0.75	0.70

To check the validity of the simulations, we also estimated information $IG\left(Y|X\right)$ of the Pelli–Robson test from its reported test–retest variabilities (TRV = 1.96 $\sigma$), with the assumption that the outcome distributions are normal and have the same TRV for all the observers. For the test, the outcome scores cover 0 to 2.25 log_10_ contrast sensitivity, with a typical TRV between 0.15 and 0.20 log10 in the normal population^55^. Therefore, $L=2.25$ log_10_ CS, $\sigma =\frac{TRV}{1.96}$ is between 0.077 and 0.10 log_10_ CS, and the expected information gain is between 2.4 and 2.8 bits. These estimated values are largely consistent with the results in Simulation 1.

For the qCSF test, we also computed information gain from two existing datasets, one with 112 subjects tested binocularly in three luminance conditions^38^ and the other with 14 eyes tested monocularly in four Bangerter foil conditions^28^. In this dataset, peak gain is between 0.92 and 2.27 log_10_ CS, peak spatial frequency is between 0.21 and 3.9 c/d, and the bandwidth is between 1.8 and 5.7 octaves. We computed the total entropy, expected residual entropy, and expected information gain based on the posterior distributions from single tests rather than distributions of test scores from repeated tests. In the qCSF test, the posterior distributions from single tests are broader than those derived from repeated tests until about 25 trials are tested and converge to those from repeated tests afterwards^34^. The expected information gain based on AULCSF is 2.0, 2.4, and 2.9 bits after qCSF test with 15, 25 and 50 trials, respectively, and the expected information gain based on CSF at six spatial frequencies is 4.1, 4.6, and 5.3 bits after qCSF test with 15, 25 and 50 trials, respectively. Again, the results from the dataset are largely consistent with those from Simulations 2.

In both simulations, CSV-1000 generated more expected information gain than the Pelli–Robson test. Scored with AULCSF or with CSF at six spatial frequencies, qCSF with 25 trials generated more expected information gain than CSV-1000. In terms of expected information gain per optotype tested, the CSV-1000 test was the most efficient in Simulation 1, and qCSF with 15 rows of trials was the most efficient in Simulation 2. The variations in efficiency were caused by different test designs and the distributions of the populations used in the study. Interestingly, the ratio of expected information gain versus the ${{\text{lo}}{\text{g}}}_{2}$ of the number of optotypes tested in qCSF was essentially constant across test lengths and was greater than the corresponding scores in Pelli–Robson in Simulation 1 and both Pelli–Robson and CSV-1000 in Simulation 2. The results suggest that the expected information gain increased linearly with the ${\text{log}}_{2}$ of the number of optotypes in the qCSF test.

## Discussion

In this study, we introduced a concept from information theory, called expected information gain (or mutual information), to quantify the amount of new knowledge that can be obtained from measurement on a population. This concept allows us to compare measurements with different dimensionalities and assess the potential advantages of new measurements that generate distinct or higher dimensional data compared to the current gold standard. Importantly, it focuses on the actual knowledge gained through the measurements, surpassing the mere quantification of measurement uncertainties.

We demonstrated two equivalent approaches for computing expected information gain:

1. *Reduction of uncertainty* This approach gauges the reduction in uncertainty regarding the “truth” (the property to be measured) after the measurement.
2. *Difference in uncertainty* The second approach calculates the difference between the uncertainty associated with all possible measurement outcomes in a population and the expected residual uncertainty after the measurement.

In both approaches, the key idea is that expected information gain quantifies the increase in knowledge achieved through measurement. This knowledge gain is greater when there is more uncertainty initially and/or when the measurement effectively reduces this uncertainty. These approaches provide a rigorous and quantitative way to assess the value of measurement in various fields of science and beyond.

As a practical application of this concept, we computed and compared expected information gain from various VA and CS tests. Our findings revealed some intriguing insights:

1. *VA tests* The ETDRS chart, the current gold standard, generated less expected information gain than the qVA test with 15 rows, indicating the potential for improved VA assessment with qVA.
2. *CS tests* The CSV-1000 test outperformed the Pelli–Robson test, but the qCSF test with 25 trials demonstrated the highest expected information gain, showcasing its advantages in assessing CS.

It’s crucial to consider the target population when comparing expected information gain, as it can vary for different populations. Our simulations demonstrated this dependence, indicating that different target populations might require different measurements, and different measurements may have advantages or disadvantages in different populations. Therefore, when comparing tests, it is crucial to consider the target population alongside the measurement instruments. In this study, we used the same target populations to compare different VA and CS tests, and consistent rank orders of the tests were obtained across the two populations.

Expected information gain isn’t limited to vision tests; it can be applied across various domains. For example, it can help compare newly developed optical coherence tomography (OCT) tests with existing ones, assess different vision tests’ abilities to classify patients, or evaluate data analytics techniques based on their capacity to reduce uncertainty^49,51^.

Expected information gain can serve as a valuable tool for objectively evaluating the new knowledge acquired through measurements. It may facilitate better comparisons and informed decision-making in diverse fields of research and data analysis.

## Figures and Tables

**Figure 7:**
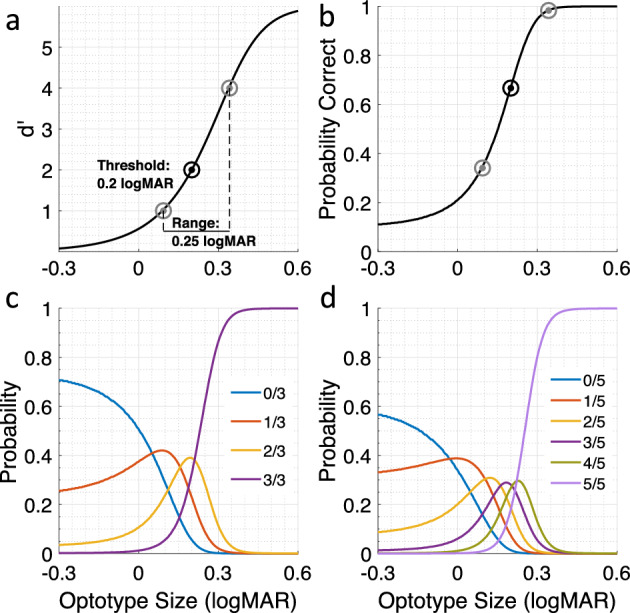
Multi-optotype acuity behavioral psychometric functions. (**a**) The dʹ psychometric function for a single optotype for an observer with VA threshold = 0.2 logMAR and VA range = 0.25 logMAR. (**b**) The percent correct psychometric function for single-optotype in a 10-AFC task for the observer in (**a**). (**c**) The multi-optotype psychometric functions of the observer in the qVA test, i.e., the probabilities of correctly identifying 0, 1, 2, or 3 of the three optotypes in a test row as functions of optotype size of the row. (**d**) The multi-optotype psychometric functions of the observer in the ETDRS test, the probabilities of correctly identifying 0, 1, 2, 3, 4 or 5 of the five optotypes presented in a test row as functions of optotype size of the row.

## Data Availability

The datasets used and/or analyzed during the current study are available from the corresponding author on reasonable request.

## Appendix 1: Proof of equivalence

It’s well-known that information gain is symmetric, that is, $\mathrm{IG}\left(\mathrm{Y}|\mathrm{X}\right)=\mathrm{IG}\left(\mathrm{X}|\mathrm{Y}\right)$. We can prove this by substituting $\mathrm{P}\left(\mathrm{x}|\mathrm{y}\right)$ with its Bayesian computation in Eq. (2):

A1 $$\mathrm{H}\left(\mathrm{X}|\mathrm{Y}\right)=-\int\limits_{\mathrm{Y}}\left(\int\limits_{\mathrm{X}}(\mathrm{P}\left(\mathrm{x}|\mathrm{y}\right){\mathrm{log}}_{2}\left(\mathrm{P}\left(\mathrm{x}|\mathrm{y}\right)\right)\mathrm{dx})\right)\mathrm{P}\left(\mathrm{y}\right)\mathrm{dy}=-\int\limits_{\mathrm{Y}}\left(\int\limits_{\mathrm{X}}\frac{\mathrm{P}\left(\mathrm{y}|\mathrm{x}\right)\mathrm{P}\left(\mathrm{x}\right)}{\mathrm{P}\left(\mathrm{y}\right)}{\mathrm{log}}_{2}\left(\frac{\mathrm{P}\left(\mathrm{y}|\mathrm{x}\right)\mathrm{P}\left(\mathrm{x}\right)}{\mathrm{P}\left(\mathrm{y}\right)}\right)\mathrm{dx}\right)\mathrm{P}\left(\mathrm{y}\right)\mathrm{dy}=\int\limits_{\mathrm{Y}}\left(\int\limits_{\mathrm{X}}\mathrm{P}\left(\mathrm{y}|\mathrm{x}\right)\mathrm{P}\left(\mathrm{x}\right){\mathrm{log}}_{2}\left(\mathrm{P}\left(\mathrm{y}\right)\right)\mathrm{dx}\right)\mathrm{dy}-\int\limits_{\mathrm{Y}}\left(\int\limits_{\mathrm{X}}\mathrm{P}\left(\mathrm{y}|\mathrm{x}\right)\mathrm{P}\left(\mathrm{x}\right){\mathrm{log}}_{2}\left(\mathrm{P}\left(\mathrm{y}|\mathrm{x}\right)\mathrm{P}\left(\mathrm{x}\right)\right)\mathrm{dx}\right)\mathrm{dy}=\int\limits_{\mathrm{Y}}\mathrm{P}\left(\mathrm{y}\right){\mathrm{log}}_{2}\left(\mathrm{P}\left(\mathrm{y}\right)\right)\mathrm{dy}-\int\limits_{\mathrm{X}}\left(\int\limits_{\mathrm{Y}}\mathrm{P}\left(\mathrm{y}|\mathrm{x}\right){\mathrm{log}}_{2}\left(\mathrm{P}\left(\mathrm{y}|\mathrm{x}\right)\right)\mathrm{dy}\right)\mathrm{P}\left(\mathrm{x}\right)\mathrm{dx}-\int\limits_{\mathrm{X}}\mathrm{P}\left(\mathrm{x}\right){\mathrm{log}}_{2}\left(\mathrm{P}\left(\mathrm{x}\right)\right)\mathrm{dx}.$$

Therefore,

A2 $$\mathrm{IG}\left(\mathrm{X}|\mathrm{Y}\right)=\mathrm{H}\left(\mathrm{X}\right)-\mathrm{H}\left(\mathrm{X}|\mathrm{Y}\right)=-\int\limits_{\mathrm{Y}}\mathrm{P}\left(\mathrm{y}\right){\mathrm{log}}_{2}\left(\mathrm{P}\left(\mathrm{y}\right)\right)\mathrm{dy}+\int\limits_{\mathrm{X}}\left(\int\limits_{\mathrm{Y}}\mathrm{P}\left(\mathrm{y}|\mathrm{x}\right){\mathrm{log}}_{2}\left(\mathrm{P}\left(\mathrm{y}|\mathrm{x}\right)\right)\mathrm{dy}\right)\mathrm{P}\left(\mathrm{x}\right)\mathrm{dx}=\mathrm{IG}\left(\mathrm{Y}|\mathrm{X}\right).$$

## Appendix 2: Multi-optotype acuity behavioral psychometric functions

From the log(*d*ʹ) acuity behavioral psychometric function for a single-optotype in Eq. (8) (Fig. 7a), we can derive the percent correct psychometric function for a single optotype for the N-alternative forced optotype identification (N-AFC) task based on signal detection theory (Fig. 7b), with N = 10 in the Snellen, ETDRS and qVA tests^21^. We then take into account of the chart design (i.e., optotype sampling with or without replacement) to derive the probability of correctly identifying different numbers optotypes in each row of a test. The multi-optotype psychometric functions are shown for the qVA (Fig. 7c) and ETDRS (Fig. 7d) tests. Specially, the three optotypes in each test row are sampled from 10 Sloan letters with replacement in the qVA test, while the five optotypes in each test row are sampled from 10 Sloan letters with replacement in the ETDRS test.
